# Correlation between BMI and Oral Health Status (DMFT, PI, mSBI, and Salivary 1,5-AG) among the Pediatric Population in Saudi Arabia: A Clinico-Biochemical Study

**DOI:** 10.3390/children9071017

**Published:** 2022-07-08

**Authors:** Sara Ayid Alghamdi, Aziza Aljohar, Basim Almulhim, Abdullah Alassaf, Smita Singh Bhardwaj, Julie Toby Thomas, Abdullah Almalki, Areej Owaid Aljuaid, Sreekanth Kumar Mallineni

**Affiliations:** 1 Department of Preventive Dental Sciences, College of Dentistry, Majmaah University, Al Majmaah 11952, Saudi Arabia; b.almulhim@mu.edu.sa (B.A.); am.assaf@mu.edu.sa (A.A.); s.bhardwaj@mu.edu.sa (S.S.B.); j.thomas@mu.edu.sa (J.T.T.); ae.almalki@mu.edu.sa (A.A.); 2 Department of Pediatric Dentistry, King Faisal Hospital and Research Center, Riyadh 11564, Saudi Arabia; aljohar@kfshrc.edu.sa; 3 Border Guard Medical Center, Riyadh 11135, Saudi Arabia; areejaljeaid@gmail.com; 4 Center for Transdisciplinary Research (CFTR), Saveetha Institute of Medical and Technical Sciences, Saveetha Dental College, Saveetha University, Chennai 600077, India

**Keywords:** children, saliva, tooth decay, body mass index, dental caries

## Abstract

The study aimed to investigate the association of varying body mass index (BMI) with oral health status among children aged 5–14 years and correlate the concentration of salivary levels of 1,5-AG with varying BMI, dental caries, and periodontal disease. This cross-sectional study was conducted on subjects aged 5 to 14 years. The children were recruited from the Pediatric Dental Clinic, College of Dentistry, Majmaah University, by convenient sampling method. Sociodemographic details and clinical parameters, including body mass index (BMI), DMFT/def (deciduous decayed tooth (d), deciduous extracted tooth (e), deciduous filled tooth (f), permanent tooth decayed (D), permanent missing tooth (M), and permanent filled tooth (F)), plaque index (PI), and modified sulcular bleeding index (mSBI), were evaluated. Salivary 1,5-anhydroglucitol (1,5-AG) was analyzed using an enzyme-linked immunosorbent assay (ELISA) for all the subjects. Statistical analyses performed using SPSS v. 27 (IBM Statistics, Chicago, IL, USA) and the Kruskal–Wallis and chi-square tests were used for comparisons. The Spearman rank correlation coefficient was used to examine the association between the study subjects’ independent variables, BMI, and caries activity. The mean def score, PI, and mSBI scores were higher in obese children. PI score, mSBI score, and salivary concentrations of 1,5-AG between the BMI categories were statistically significant (*p* < 0.001). The study emphasizes promoting preventive oral health regimes, health awareness campaigns, and nutritional educational programs among the pediatric population.

## 1. Introduction

Good oral health reflects an individual’s positive well-being and ability to adjust to all the physiological and psychological changes throughout his or her lifetime. Dental practice often sets up an ideal environment for motivating children and young adults to carry out independent self-care dental hygiene procedures, maintain a healthy diet, and initiate the preventive process [1]. The current COVID-19 pandemic has increased the prevalence of chronic oral diseases, such as dental caries and gingivitis, despite being highly preventable among the pediatric population around the globe. This surge could be related to the fact that pediatric dentists focused primarily on emergency interventions, and specific dental protocols were followed to control the spread of viral transmission [2]. Children are deprived of a healthy diet and access to oral health care facilities, further getting accustomed to electronic gadgets, mental stress, and social isolation [3].

Dental caries manifests as demineralization of the complex tooth structure, resulting in deep cavitation and toothache if allowed to progress. The increased prevalence of dental caries among the Saudi Arabian pediatric population is more associated with poor brushing habits, snacking between meals, low consumption of fruits, and frequent consumption of soft drinks and flavored milk, irrespective of socioeconomic factors, as seen in low-income and middle-income countries [4]. This could reflect on the child’s overall well-being and behavioral pattern, negatively impacting school performance and exacerbating social inequalities. From a dental practitioner’s perspective, it makes solid sense to initiate an oral health improvement program and monitor the effective implementation of oral disease prevention measures [5].

Over the past decade, the increasing burden of overweight and obesity among Saudi children and adolescents has profoundly impacted their physical health and social and emotional well-being [6]. WHO reports state that childhood obesity is linked to various comorbidities, including metabolic, cardiovascular, orthopedic, neurological, hepatic, pulmonary, renal, and periodontal disease [7,8]. Globally, periodontal disease is considered the 11th most prevalent condition [9]. A cross-sectional study conducted among Saudi Arabian high school children demonstrated a prevalence of gingivitis at 65%, with the primary etiology being attributed to poor oral hygiene practices [10]. Obesity, dental caries, and periodontal disease share common risk factors, such as increased sugar consumption, dental biofilm, socioeconomic factors, and behavioral and genetic predisposing factors [11]. Despite conflicting reports, Al-Ansari et al. [12] demonstrated that obese children are 2.21 times more likely to have been affected by dental caries than children with a low caries experience.

A longitudinal southern Chinese study revealed that poor dental health care among overweight/obese individuals with a higher body mass index in the teenage years of 15 was associated with periodontal inflammation at the age of 18, building the need for public health to prioritize improving knowledge on oral hygiene practices and healthy eating habits among secondary school students [13]. Reduced salivary flow rate, higher visual plaque index (VPI), and bleeding on probing (BOP) were observed among obese 14-year-old patients with dental caries [14]. Oral health education and reinforcement of oral health practices in children are quite challenging for dental clinicians [15]. Noninvasive techniques are becoming popular in the present era, and clinicians and researchers are preferring to use saliva samples over blood samples [16]. Saliva has added advantages over blood as it hardly coagulates, is simple to collect, and is easy to transport and store for further analysis [17].

1,5-Anhydroglucitol (1,5-AG), a naturally occurring monosaccharide, is derived from diet and absorbed in the gut. Patients with standard glycemic control maintain constant plasma and salivary concentrations of 1,5-AG. It has been considered as an emerging marker for glycemic control. It has been found to have an inverse association with blood sugar concentration [18]. Its levels vary depending on the type and proportion of the consumed carbohydrates [19]. Studies have found a significant difference in the levels of salivary 1,5-AG among high-caries-risk and caries-free children [18,19]. Syed et al. [20] demonstrated a strong negative correlation between salivary levels of AG and the high-caries-risk group. This could serve as an early indicator of caries status in children prior to tactile detection. There is still limited substantive literature on salivary diagnostics in the early identification of oral disease among children. Public health-care sectors should prioritize screening for early diagnosis and oral monitoring conditions, further implementing preventive practices by educating the parents on the importance of a healthy diet regimen and lifestyle pattern for better oral health. Henceforth, the objective of this study was to investigate the association of varying body mass index (BMI) with oral health status among children aged 5–14 years and to correlate the concentration of salivary levels of 1,5-AG with BMI dental caries and periodontal disease.

## 2. Materials and Methods

Ethical approval for this study was granted by the Institutional Review Board of Majmaah University (Ethics Number: MUREC- August.25/COM-2021 I 2-1), following the principles stated in the Declaration of Helsinki (25/08/2021). Written consent was obtained from the parents/guardians of all the subjects, along with verbal consent from all the study subjects. A convenient sample of children attending the Pediatric Dental Clinic, College of Dentistry, Majmaah University, was collected between 1 September 2022 and 20 September 2021. A minimum sample size of 95 children was included in the study, considering the statistical power of 80% from the study reported by Al-Ansari et al. [12]. Healthy children aged 5–14 years from the regular patients’ pool were included in the study. Children who were attending an emergency treatment, were undergoing orthodontic treatment, were medically compromised, were using any medications for the past 6 months, and had arrested carious lesions were excluded from the study. The participation of the children in the interview session and clinical examinations was conducted as per the COVID-19 protocol standards set by the World Health Organization (WHO). The parents of the children were requested to respond to the interview session conducted by a single examiner, who recorded the sociodemographic details digitally, including age, gender, education levels, previous dental and medical history, diet histories such as number of meals and consumption of sugar-containing snack or beverages per day, and oral hygiene practices.

Body height and body weight were measured for all the subjects by one trained and calibrated examiner following the standardized protocol recommended by Lohman et al. using a physician’s scale and stadiometer [21]. Body mass index (BMI) was evaluated by dividing the weight (kg) by the square of the height (m^2^). All the subjects were categorized based on the BMI score, which included underweight (<18 kg average), normal weight (18.5–24.9 kg/m^2^), overweight (25–29.9 kg/m^2^), and obese (≥30 kg/m^2^) [22].

Two calibrated examiners performed clinical assessments using a dental mirror, community periodontal index (CPI) probe, dental explorer, gauze, artificial light source, and plaque disclosing agent. Intra- and interinvestigator reliabilities were evaluated to be 0.89 and 0.83 using the kappa coefficient. Dental caries and periodontal health were assessed using the decayed, missing, and filled teeth index for permanent (DMFT) and deciduous teeth (dmf/def index), modification of Quigley–Hein plaque index, and modified sulcular bleeding index (mSBI) [23,24,25]. The periodontal pocket depth and clinical attachment loss were used to assess periodontal health [26].

The plaque index (PI) was evaluated using a two-tone disclosing agent with a microbrush on the tooth surfaces. The tooth surfaces were rinsed after 2 min for 30 s with the help of water spray and high-volume suction. The color-stained plaque was assigned a score in the range of 1–5 based on these criteria: 0 = no plaque, 1 = separate flecks of plaque at the cervical margin of the tooth, 2 = a thin continuous band of plaque (up to 1 mm) at the cervical margin of the tooth, 3 = a band of plaque wider than 1 mm but covering less than one-third of the crown of the tooth, 4 = plaque covering at least one-third but less than two-thirds of the crown of the tooth, and 5 = plaque covering two-thirds or more of the crown of the tooth.

mSBI and periodontal pocket depth (PPD) were evaluated using a sterile CPI periodontal probe [27]. An mSBI score ranging from 0 to 3 was assigned to each facial and lingual nonrestored surface of all the teeth according to the following criteria: 0 = no bleeding when the periodontal probe is passed along the gingival margin, 1 = isolated bleeding spots visible, 2 = blood forms a confluent red line on the gingival margin, and 3 = heavy or profuse bleeding. The average score for all the teeth was taken for data analysis. A 5 mL of saliva was collected 2 h after breakfast by the spitting method from all the children in the study sample [28]. The children were instructed to spit into sterile graduated 5 mL polypropylene vials after 30 s. The principal investigator labeled the samples blinded to the subjects’ sociodemographic and clinical assessment records. All the samples were stored at −80 °C until further analysis.

### 2.1. Quantitative Estimation of Salivary 1,5-AG Using Enzyme-Linked Immunosorbent Assay (ELISA)

An ELISA kit for 1,5-AG (CEB046Ge, Cloud-Clone Corp., Katy, TX, USA) was used with minor modifications of the protocol provided by the manufacturer. The stored saliva was thawed and centrifuged (Kenley, London, UK, rotor radius: 7 cm) at 3500 rpm for 10 min to remove proteins; the clear supernatant was collected to estimate 1,5-AG levels. To make the calculation more accessible, the OD value of the standard (X-axis) was plotted against the standard’s concentration (Y-axis) log. The best-fit straight line was drawn through the standard points, as determined by regression analysis, using the plotting software CurveExpert version 2.6.4. The concentration of 1,5-AG in the saliva samples was determined from this curve.

### 2.2. Statistical Analysis

All the data obtained were entered into MS Excel (Microsoft 2019, Redmond, WA, USA) for further statistical analysis with SPSS v. 27 (IBM Statistics, Chicago, IL, USA). The Kolmogorov–Smirnov test showed that the data distribution of 1,5-AG violates the normality assumption, and we opted for nonparametric statistical methods to analyze the collected data on study variables. The Kruskal–Wallis and chi-square tests were applied to compare all the study subjects’ BMI scores and clinical parameters. The Spearman rank correlation coefficient was used to examine the association between the study subjects’ independent variables, BMI, and caries activity. Descriptive statistics, such as frequency distributions, percentages, means, and standard deviations, were used to summarize the data collected. A calculated *p*-value less than 0.05 is considered statistically significant.

## 3. Results

**Figure 1:**
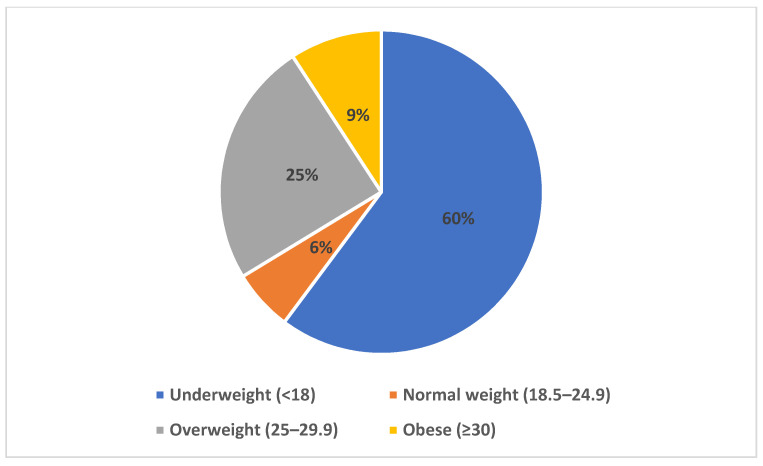
Distribution of participants according to four categories of BMI.

**Figure 2:**
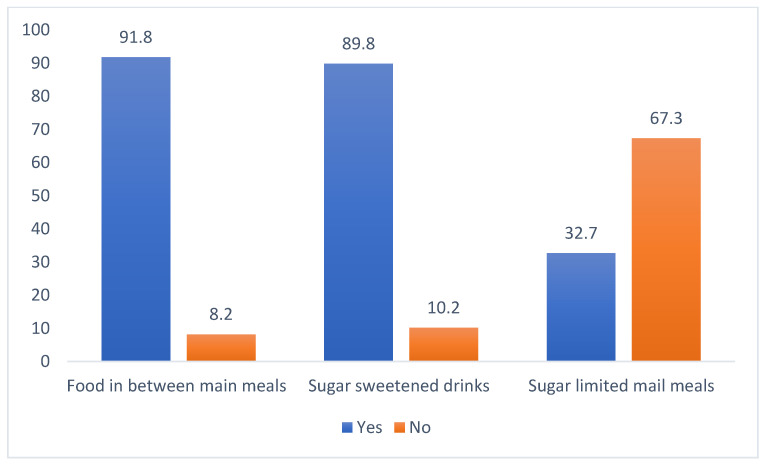
Frequency of food and sugar intake among the study participants.

The mean age of the children that participated in the study sample was 8.5 (2.0) years. The mean BMI of the subjects was 18.8 (7.08). The mean def score was 3.0 (3.4). This was found to be higher when compared with the mean DMFT score of 2.9 (2.7). The mean number of decayed teeth ‘d’ scores of the participants was 5.7 (2.4), while the number of permanent decayed ‘D’ scores was 2.5 (2.2). The mean PI and mSBI scores were 2.1 (1.2) and 0.6 (0.6). The mean biochemical salivary levels of 1,5-AG was 1.9 (1.6) μg/mL (Table 1). The majority of the participants reported an average eating speed (50.0%), while 20.4% reported eating fast. The majority of the subjects (86%) had no family history of periodontal disease or diabetes mellitus. About 95% of the participants used a brush, and 80.6% reported brushing once daily. Most of the subjects (67.3%) visited the dentist once a year (Table 2). Most of the subjects did not have any physical activity as part of their daily routine. Figure 1 shows the distribution of the participants based on four categories of BMI, and Figure 2 shows the frequency of food and sugar intake among the subjects. The majority of the children (91.8%) reported having food between main meals. About 89.8% reported having sweetened drinks between their main meals, and 67.3% of the subject’s sugar consumption was limited to the main meals.

The relationship between BMI and clinical parameters among the study subjects is shown in Table 3). The mean def score was higher among the participants belonging to the obese BMI category. The mean DMF score was higher among the participants belonging to the normal BMI category. However, these differences were not found to be statistically significant. The PI and mSBI scores were high among the normal and overweight BMI categories. The salivary 1,5-AG levels were highest among the obese participants. The difference in the PI score, mSBI score, and salivary concentrations of 1,5-AG between the BMI categories was statistically significant (*p* < 0.001).

The association between the independent variables, BMI, and caries activity of these children is shown in Table 4. The BMI score was positively correlated with PI, mSBI, and salivary 1,5-AG levels. This indicates that as BMI scores increased, the PI, mSBI, and salivary 1,5-AG levels were also increased (*p*-value = 0.003, 0.001, and 0.001, respectively). The mean DMF scores were positively correlated with the PI scores (*p*-value = 0.007). The PI scores were positively correlated with mSBI, and the results were statistically significant (*p* < 0.001). The association between sociodemographic characteristics and BMI in shown in Table 5. There was a significant association between sugar intake between main meals and obesity (*p*-value = 0.02). The majority of the participants who reported eating at an average speed (52.8%) belonged to the nonobese category.

## 4. Discussion

The study population comprised 95 pediatric patients aged 5–14 years. Based on the BMI score, about 60% belonged to the underweight category, 9.2% were found to be obese, and 6% were overweight subjects. The study’s findings were in accordance with the observations of Hijji et al. [29], who reported an increasing prevalence of underweight status among the adolescent group in Saudi Arabia. Studies have also reported that the overall growing prevalences of childhood overweight and obesity in Riyadh were about 13.4 and 18.2 [5]. The emergence of the global pandemic was thought to have hardly affected the childhood population, despite indirectly creating a drastic change in behavioral lifestyle patterns among the pediatric population. Various studies have demonstrated the unprecedented consequences of childhood home confinement; certain studies have stated that deprivation of oral health care facilities, unhealthy diet patterns, sedentary lifestyle, and decreased physical activity resulted in childhood obesity in high-income and developed countries. In contrast, other studies reported underweight and malnutrition among the population belonging to low-income countries. This dual burden of overweight and underweight among the pediatric population could negatively influence their overall health status [30]. The salivary biomarker 1,5-AG has been used as a noninvasive marker for screening at-risk diabetic patients. Juraschek et al. [19] concluded that reducing the type and amount of dietary carbohydrates reduced 1,5-AG concentrations in a population without diabetes. In the present study, concentrations of salivary 1,5-AG were found to be increased in the obese category (*p* < 0.05). Yeong et al. [31] demonstrated that overweight/obese subjects had greater 1,5-AG levels and lower glycated hemoglobin levels (HbA1c) than the diabetes group.

Caries estimation in this study population was performed by evaluating the def and DMF index scores [24]. Summarizing the results from descriptive statistics of the study, it was found that the def score was found to be greater than the mean DMF score, indicating that deciduous dentition was more affected compared with permanent dentition. The mean deciduous decay score (d) of 5.67 (2.37) was comparatively higher than the mean permanent decay score (D) of 2.53 (2.19). The findings in the study are consistent with those in a study by Farooqi et al. [32], where the mean dmft value was 3.66 (3.13) and the mean DMFT value was 1.94 (2.0). Data reports stated a dramatic rise in the prevalence of dental caries of about 60–85% among the pediatric population with a mean def score of 1.4 and a DMFT score of 1.72–2.66 [33]. There is a necessity to alert public health workers to concentrate more on educating and creating awareness among parents of the importance of regularizing the need for dental hygiene after feeding and promoting preventive measures to arrest the progression of dental caries [34,35].

The sociodemographic characteristics observed in this study’s caries activity were related to inadequate oral hygiene practices, infrequent dental visits, increased sugar content, and unhealthy dietary pattern. These findings are consistent with those of an investigation conducted among high school students by Fagerberg et al. [36]. The authors reported a conclusive remark on the association between the objectively measured eating rate with students’ weight status. They found that students with fast eating rates consumed more food during school lunch compared with students with a slow eating rate.

Investigative research has been focusing on specific salivary biomarkers that could serve as a potential tool for early diagnosis and preventing the progression of dental caries. A weak negative correlation between caries index and salivary 1,5-AG was observed in this study. In contrast, an Arabian recent survey [20] demonstrated a strong negative correlation between salivary 1,5-AG and the caries active group and the authors concluded that salivary 1,5-AG can act as an indicator of caries status in children. AG, a metabolically inert monosaccharide, inhibits the growth of cariogenic bacteria, thus decreasing the caries activity in the oral environment. Nonetheless, the development of cariogenic bacteria favors increased fermentation of the saccharide substrate for cellular metabolism, thus reducing the salivary 1,5-AG levels [19].

Gingivitis rarely progresses to periodontitis in children. Therefore, none of the study subjects demonstrated clinical loss of attachment. In the present study, the mean scores of PI and mSBI were 2.11 (1.21) and 0.65 (0.65). A cross-sectional study of high school children observed a high prevalence of gingivitis mainly due to poor oral hygiene practices [9]. So far, no studies have reported the periodontal health status among children aged 5–14 years. On analyzing the degree of association of chronic oral diseases with BMI scores, it was found that obese children demonstrated a higher def score. Still, the findings were not found to be statistically significant. This could be probably due to an inadequate sample size in different BMI categories. The result was consistent with other observational studies in the Saudi Arabian population, where the caries index and obesity relationship did not infer any statistically significant impact [37]. A cross-sectional study [11] observed that obese participants had a higher mean DMFT score than nonobese participants. This infers the need to create awareness among the child population to modify their lifestyle and behavioral patterns concerning intake of healthy balanced nutrition, indulging more in physical activity, and practicing regular oral health care.

Analysis by the Kruskal–Wallis test demonstrated a statistically significant (*p* < 0.05) positive correlation between the PI, mSBI, and higher BMI scores. These findings were in accordance with a recent Arabian study [38] that concluded that obese subjects are more prone to gingival diseases than nonobese subjects. Oral bacteria could contribute to increased metabolic deficiency, leading to unwanted weight gain irrespective of calorie consumption, diet, or exercise. An increase in bleeding observed in obese children could probably be due to an increased level of C-reactive protein and fibrinogen produced by the liver to initiate the chronic subclinical systemic inflammatory response. Furthermore, the prevalence of periodontal disease decreased with increased physical activity and was directly proportional to the frequency of activity. Oral bacteria could increase appetite following weight gain, alter tissue resistance to insulin by increasing the levels of proinflammatory cytokines, and reduce adiponectin levels, disrupting its modulatory role in periodontal health [39]. Saliva, being a natural reservoir, can harbor various biochemical molecules, which can mitigate triggering inflammation and cause deterioration of the tooth-supporting tissue [40,41]. Goodson et al. [42] identified multiple metabolites in saliva at different levels of obesity and gingivitis. 1,5-AG, being a diet-derived monosaccharide, excess in calorie consumption is positively associated with obesity. Statistical analysis of the study data demonstrated a significant positive correlation between the BMI scores and PI, mSBI, and salivary concentrations of 1,5-AG. Statistically effective results were observed for chronic oral diseases, such as dental caries (*p* < 0.007) and gingivitis (*p* < 0.001), with PI. Further longitudinal studies are warranted to detect the role of 1,5-AG in dental caries and gingivitis in correlation with various dietary patterns. The association of 1,5-AG with dental caries and gingivitis was not statistically evident in this study. This could be due to the premises of the cross-sectional study design and the insufficient expression of the biomarker in saliva. To accomplish sustainable improvements in children’s health, the nation must emphasize the need to create awareness among the pediatric population of the predisposing factors affecting health and develop effective strategies for early diagnosis, monitoring, and maintenance of global oral health.

## 5. Conclusions

Within the study’s limitations, the mean def, PI, and mSBI scores were higher in obese children. The PI score, mSBI score, and salivary concentrations of 1,5-AG between the BMI categories were statistically significant. The study suggests that salivary 1,5-AG levels could be used as a noninvasive indicator to assess the obesity status of children.

## Figures and Tables

**Table 1:** Details of the study sample.

Variables	Mean ± S.D.
Age	8.59 ± 2.06
Height	127.0 ± 16.20
Weight	30.75 ± 13.78
Body mass index (BMI)	18.86 ± 7.08
Deciduous decayed tooth (d)	5.67 ± 2.37
Deciduous extracted tooth (e)	1.50 ± 0.57
Deciduous filled tooth (f)	1.78 ± 0.97
def score	3.00 ± 3.35
Permanent tooth decayed (D)	2.53 ± 2.19
Permanent missing tooth (M)	0.93 ± 0.73
Permanent filled tooth (F)	1.68 ± 1.35
DMF score	2.90 ± 2.46
Plaque index (PI)	2.11 ± 1.21
Modified sulcular bleeding index (mSBI)	0.65 ± 0.65
Salivary 1,5-AG level in μg/mL	1.88 ± 1.56

DMF = Decayed missing, and filled teeth.

**Table 2:** Characteristics of the sample.

Characteristics of the Study Subjects		Frequency (*n* = 95)	Percentage (%)
Eating speed	Very slow	7	7.1
	Slow	19	19.4
	Ordinary	49	50.0
	Fast	20	20.4
	Very fast	3	3.1
Family history of periodontal disease	Yes	13	13.3
	No	85	86.7
Family history of diabetes mellitus	Yes	13	13.3
	No	85	86.7
Use of brush in routine Daily oral hygiene	Yes	94	95.1
	No	4	4.9
Brushing frequency	No	2	2.0
	Occasional	1	1.0
	Once	79	80.6
	Twice	16	16.3
Frequency of dental visit	Never	9	9.2
	Occasionally	22	22.4
	Once a year	66	67.3
	More than once a year	1	1.0
Physical exercise in their daily routine	No activity	9	9.2
	Sometimes	87	88.8
	Regular	2	2.0

**Table 3:** Relationship between BMI and clinical parameters among the study participants.

Variables	BMI	Mean	Std. Deviation	Std. Error	*p*-Value
Def	Underweight	3.18	3.65	0.48	0.748
	Normal weight	2.72	3.06	0.61	
	Overweight	1.50	1.97	0.80	
	Obese	3.55	2.87	0.95	
	Total	3.00	3.35	0.33	
DMF	Underweight	2.84	2.53	0.33	0.066
	Normal weight	3.64	2.41	0.48	
	Overweight	1.00	2.00	0.81	
	Obese	2.55	1.87	0.62	
	Total	2.90	2.46	0.24	
PI	Underweight	1.74	1.03	0.13	0.003 *
	Normal weight	2.84	1.37	0.27	
	Overweight	2.33	1.03	0.42	
	Obese	2.33	1.11	0.37	
	Total	2.11	1.21	0.12	
mSBI	Underweight	0.39	0.61	0.08	0.001 *
	Normal weight	0.96	0.61	0.12	
	Overweight	1.33	0.51	0.21	
	Obese	1.00	0.00	0.00	
	Total	0.65	0.65	0.06	
Salivary 1,5-AG levels (μg/mL)	Underweight	1.41	1.23	0.16	0.001 *
	Normal weight	2.07	1.10	0.22	
	Overweight	1.70	1.40	0.57	
	Obese	4.52	2.14	0.71	
	Total	1.88	1.56	0.15	

Body mass index (BMI), deciduous decayed tooth (d), deciduous extracted tooth (e), deciduous filled tooth (f), permanent tooth decayed (D), permanent missing tooth (M), permanent filled tooth (F), plaque index (PI), modified sulcular bleeding index (mSBI), Kruskal–Wallis test, * *p*-value < 0.05 statistically significant.

**Table 4:** Association between independent variables, BMI, and caries activity.

Variables		Def	DMF	PI	mSBI	Salivary 1,5-AG
BMI	Correlation coefficient	−0.089	0.122	0.222	0.441	0.402
	Sig. (2-tailed)	0.383	0.232	0.028 *	0.001 *	0.001 *
	*n*	98	98	98	98	98
def	Correlation coefficient		−0.468	−0.075	0.107	−0.048
	Sig. (2-tailed)		0.001 *	0.460	0.295	0.637
	*n*		98	98	98	98
DMF	Correlation coefficient			0.269	0.095	−0.081
	Sig. (2-tailed)			0.007 *	0.350	0.426
	*n*			98	98	98
PI	Correlation coefficient				0.548	0.159
	Sig. (2-tailed)				0.001 *	0.119
	*n*				98	98
mSBI	Correlation coefficient					0.128
	Sig. (2-tailed)					0.209
	*n*					98

Body mass index (BMI), deciduous decayed tooth (d), deciduous extracted tooth (e), deciduous filled tooth (f), permanent tooth decayed (D), permanent missing tooth (M), permanent filled tooth (F), plaque index (PI), modified sulcular bleeding index (mSBI), Spearman correlation. * Correlation is significant at the 0.05 level.

**Table 5:** Association between sociodemographic characteristics and BMI.

Socio Demographic Charters Tics	Options	BMI		Overall	*p*-Value
		Not Obese	Obese		
Eating speed	Very slow	7 (7.9)	0 (0)	7 (7.1)	0.001 *
	Slow	18 (20.2)	1 (11.1)	19 (19.4)	
	Ordinary	47 (52.8)	2 (22.2)	49 (50)	
	Fast	17 (19.1)	3 (33.3)	20 (20.4)	
	Very fast	0 (0)	3 (33.3)	3 (3.1)	
Food intake between main meals	Yes	81 (91)	9 (100)	90 (91.8)	0.348
	No	8 (9)	0 (0)	8 (8.2)	
Intake of sweetened drinks between meals	Yes	81 (91)	7 (77.8)	88 (89.8)	0.211
	No	8 (9)	2 (22.2)	10 (10.2)	
Sugar intake limited to main meals	Yes	32 (36)	0 (0)	32 (32.7)	0.028 *
	No	57 (64)	9 (100)	66 (67.3)	
Family history of periodontal disease	Yes	12 (13.5)	1 (11.1)	13 (13.3)	0.842
	No	77 (86.5)	8 (88.9)	85 (86.7)	
Family history of diabetes mellitus	Yes	12 (13.5)	1 (11.1)	13 (13.3)	0.84
	No	77 (86.5)	8 (88.9)	85 (86.7)	
Brush usage	No	4 (4.5)	0 (0)	4 94.1)	0.516
	Yes	85 (95.5)	9 (100)	94 (95.9)	
Brushing frequency	No	2 (2.2)	0 (0)	2 (2)	0.497
	Sometimes	1 (1.1)	0 (0)	1(1)	
	Once	70 (78.7)	9 (100)	79 (80.6)	
	Twice	16 (18)	0 (0)	16 (16.3)	
Previous dental visit	Rarely	8 (9)	1 (11.1)	9 (9.2)	0.384
	Less often	18 (20.2)	4 (44.4)	22 (22.4)	
	Once a year	62 (69.7)	4 (44.4)	66 (67.3)	
	More than once	1 (1.1)	0 (0)	1 (1)	

Chi-square test; * *p*-value < 0.05 is considered statistically significant.

## Data Availability

The data will be available upon request to the correspondence author.
